# Impacts of Co-Solvent Flushing on Microbial Populations Capable of Degrading Trichloroethylene

**DOI:** 10.1289/ehp.6937

**Published:** 2004-12-08

**Authors:** Vijayalakshmi Ramakrishnan, Andrew V. Ogram, Angela S. Lindner

**Affiliations:** ^1^Department of Soil and Water Sciences, and; ^2^Department of Environmental Engineering Sciences, University of Florida, Gainesville, Florida, USA

**Keywords:** co-solvent flushing, methanotrophs, particulate methane monooxygenase, perchloroethylene, 16S rDNA, soluble methane monooxygenase, trichloroethylene

## Abstract

With increased application of co-solvent flushing technologies for removal of nonaqueous phase liquids from groundwater aquifers, concern over the effects of the solvent on native microorganisms and their ability to degrade residual contaminant has also arisen. This study assessed the impact of ethanol flushing on the numbers and activity potentials of trichloroethylene (TCE)-degrading microbial populations present in aquifer soils taken immediately after and 2 years after ethanol flushing of a former dry cleaners site. Polymerase chain reaction analysis revealed soluble methane monooxygenase genes in methanotrophic enrichments, and 16S rRNA analysis identified *Methylocystis parvus* with 98% similarity, further indicating the presence of a type II methanotroph. Dissimilatory sulfite reductase genes in sulfate-reducing enrichments prepared were also observed. Ethanol flushing was simulated in columns packed with uncontaminated soils from the dry cleaners site that were dosed with TCE at concentrations observed in the field; after flushing, the columns were subjected to a continuous flow of 500 pore volumes of groundwater per week. Total acridine orange direct cell counts of the flushed and nonflushed soils decreased over the 15-week testing period, but after 5 weeks, the flushed soils maintained higher cell counts than the nonflushed soils. Inhibition of methanogenesis by sulfate reduction was observed in all column soils, as was increasing removal of total methane by soils incubated under methanotrophic conditions. These results showed that impacts of ethanol were not as severe as anticipated and imply that ethanol may mitigate the toxicity of TCE to the microorganisms.

Co-solvent flushing, also known as *in situ* flushing, is a technology that has recently been considered for removal of light and dense nonaqueous phase liquids (LNAPLs and DNAPLs, respectively) from ground-water aquifers. Originally developed by the petroleum industry for enhanced oil recovery (Lake 1989), this method involves *a*) injection of co-solvent such as alcohol or surfactant into the source zone area of an NAPL plume; *b*) partitioning of the contaminant into the co-solvent–groundwater phase; and *c*) its recovery and *ex situ* separation from the co-solvent–groundwater mixture, which is subsequently recycled into the aquifer for capture of additional contaminant [U.S. Environmental Protection Agency (U.S. EPA) 2002]. This method promises to be superior to other technologies used for contaminant removal from aquifers because it is simple in concept, effective, and does not require removing contaminated soils (Falta et al. 1998; Rao et al. 1997).

Various bench and field studies have reported successful removal of LNAPLs and DNAPLs using this method (Brandes and Farley 1993; Imhoff et al. 1995; Jawitz et al. 2000; McCray and Brusseau 1998; Rao et al. 1997), and to date 16 Superfund sites reportedly have been successfully treated by this method (U.S. EPA 2002). Jawitz et al. (2000) recently reported an *in situ* flushing pilot study using ethanol as a co-solvent to remove perchloroethylene (PCE) DNAPLs from a shallow, unconfined aquifer at a former dry cleaners site in Jacksonville, Florida, USA. Flushing of 34 kL of a 95% ethanol/5% water mixture over a 3-day period (an equivalent of two pore volumes) resulted in 65% removal of the 68 L of PCE originally present, and these authors concluded that continued alcohol flushing would have resulted in greater NAPL removal effectiveness.

The presence of high concentrations of ethanol in an aquifer may result in significant changes in numbers and activities of microorganisms after the bulk of the contaminant has been removed. High concentrations of ethanol and certain detergents are toxic to many microorganisms. Studies have shown that such stress tends to lower the diversity in microbial communities, which are subsequently less capable of dealing with further environmental fluctuations (Atlas and Bartha 1987). Although previous work has reported positive effects of low concentrations of ethanol as an electron donor in reductive dehalogenation processes (Gibson and Sewell 1992), no study has directly observed changes in microbial populations at a site after ethanol flushing, particularly in terms of their potential to degrade residual contaminant over time. The broad objective of this study was to assess the effects of ethanol flushing over time on numbers and activity of potential PCE-and trichloroethylene (TCE)-degrading microbial populations present in soil from the former Sages Dry Cleaners site (henceforth referred to as the Sages site).

## Materials and Methods

**Figure 1:**
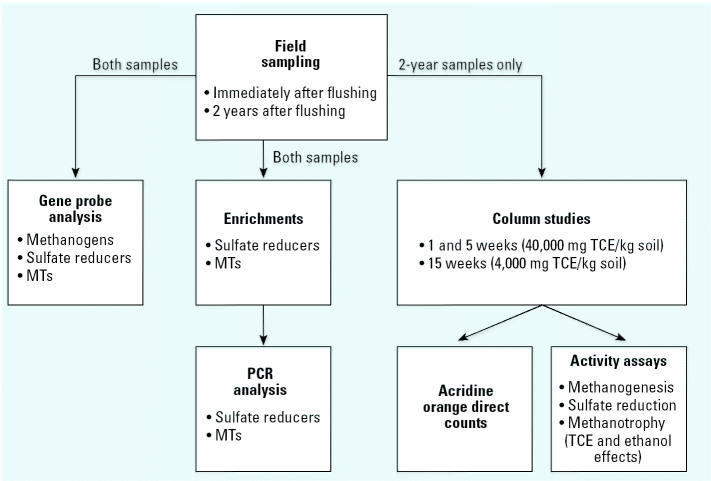
Schematic of experimental methods used in this study. Samples taken both immediately after and 2 years after ethanol flushing were used in the gene probe analysis and enrichment experiments. Only 2-year postflushing samples from C-34, C-35, and C-37 were used in the column studies.

Specific objectives of this study were the following: *a*) to obtain samples from the Sages site after ethanol flushing treatment; *b*) to assess the shifts in methanogenic, sulfate-reducing, and methanotrophic bacteria, all known to transform TCE, before and after ethanol flushing using gene probe analysis on the soil samples; *c*) to verify the presence of potential TCE-degrading bacteria by enriching for these microorganisms from the soil samples and identifying them using polymerase chain reaction (PCR) analysis; and *d*) to simulate ethanol flushing in column studies to enable determination of the impacts of ethanol and TCE on bacterial counts and activity potentials. Figure 1 provides an overview of the methods performed in this study. Each method is described in more detail below.

### Soil samples.

All samples were collected by Levine-Fricke-Recon, Inc. (Tallahassee, FL) from multilevel sampling locations at the Sages site, where ethanol flushing treatment was performed in August 1998. The locations of the seven recovery wells that surrounded the three injection wells were selected to be just outside the perimeter of the initial estimated horizontal extent of the PCE source zone as described by Jawitz et al. (2000), and all samples were removed at various distances from the recovery well zone. Immediately after ethanol flushing, soil samples were removed from three monitoring well (MW) sites, designated as MW-8, MW-9, and MW-11, located approximately 51, 26, and 97 feet, respectively, from the closest recovery well. Samples were also removed 2 years after flushing from seven locations, designated as C-31, C-32, C-33, C-34, C-35, C-36, and C-37. C-36 was located closest to the recovery well area at approximately 17 feet, and C-34 was farthest from the recovery well area at approximately 109 feet. Detailed descriptions of the Sages site contaminant plume and placement of injection, recovery, and monitoring wells can be found in Jawitz et al. (2000), and Ramakrishnan (2002), Sillan (1999). All samples were taken approximately 8–9 m below ground surface. Soil samples were collected in sterile glass jars, immediately sealed, and placed on ice. Upon arrival in the laboratory in Gainesville, Florida, soil samples were manually homogenized using a sterile spatula. An aliquot of each sample for immediate use was stored at 4°C, and the remainder of the soil samples was stored at −80°C.

Soil samples had a particle size distribution of fine to very fine sand, an average moisture content of 19.7%, and an average organic carbon content of 1.7% (Sillan 1999). The average pH of the soils used in this study ranged from 4.2 to 7.2; sulfate concentrations in these soils, measured by the University of Florida Extension Soils Testing Laboratory in Gainesville, Florida, ranged from 1.4 to 2.9 mg/L.

### Enrichments of specific TCE degraders from Sages site soil.

Because increased concentrations of methane and TCE and decreased concentrations of sulfate were observed at the Sages site subsequent to ethanol flushing, there was strong indication of the presence of methanogenic and sulfate-reducing bacteria and even possibly methane-oxidizing bacteria, given the reported aerobic preflushing conditions (Mravik et al. 2003). Methanogens and sulfate reducers are capable of anaerobic reductive dehalogenation of PCE and TCE (e.g., Bagley and Gossett 1990; DiStefano et al. 1992; Freedman and Gossett 1989) and that MTs are capable of oxidizing TCE (e.g., Dispirito et al. 1992; Henry and Grbić-Galić 1990). Therefore, enrichments of sulfate-reducing and methanotrophic bacteria were attempted as a means of verifying their presence at the site. Because of the difficulty experienced in enriching for methanogenic bacteria, only activity assays were performed for these bacteria, as described below.

### Enrichments and culturing of sulfate-reducing bacteria.

Five grams (wet weight) of soil was added to a 120-mL serum vial containing 45 mL basal carbonate yeast extract trypticase (BCYT) medium prepared anaerobically, and the headspace was flushed with N_2_:CO_2_ (70:30 v/v). This mixture was amended with acetate or lactate (20 mM), ferrous sulfate (2 mM), and sodium cysteine (0.5 g/L) (Widdel and Bak 1992). On formation of sulfide (as indicated by black precipitate), transfers were made on a regular basis to fresh BCYT medium with sodium sulfate (20 mM) instead of ferrous sulfate.

### Enrichments and culturing of methanotrophic bacteria.

Ten grams (wet weight) of soil was mixed with 50 mL nitrate mineral salts medium (NMS) (Whittenbury et al. 1970) in a 250-mL Erlenmeyer flask. The flasks were sealed with a rubber stopper threaded with glass wool–filled tubing to allow removal of headspace air, using a vacuum pump and subsequent filling with 99.99% methane (Strate Welding, Jacksonville, FL), as previously described (Lindner et al. 2000). All enrichments were prepared in triplicate using 20% methane (v/v) in the headspace and incubated at 30°C with shaking at 250 rpm. Transfers of 10–14% inoculum (v/v) to fresh liquid NMS medium were prepared weekly along with streaking on solid plates of carbon-free Bacto agar (Difco, Detroit, MI) and NMS that were stored in an airtight desiccator filled with methane:air (30% v/v) at 30°C. Cultures were periodically streaked on nutrient agar plates to assess growth characteristics of heterotrophic bacteria.

A qualitative assay was performed using naphthalene and tetrazotized *ortho*-dianisidine to detect activity of soluble methane monooxygenase (sMMO) in the enriched methanotrophic-mixed cultures, as previously described (Bowman et al. 1993; Lindner et al. 2000).

### Ethanol-flushing simulation in columns.

Laboratory-scale vertical upward flow soil columns were constructed using custom-made glass columns 5 cm long and 2.5 cm inner diameter (Kontes, Vineland, NJ) and 50 g (wet weight) of soil samples taken from an uncontaminated portion of the Sages site (2 years post-flushing). Three sets of columns were constructed and run over three time periods (1 week, 5 weeks, and 15 weeks) before being sacrificed for subsequent testing. Each set contained controls with TCE only (no ethanol) and duplicate columns containing TCE and flushed with ethanol. An additional control with ethanol treatment only was included in the 15-week column studies.

Because of the relatively small amount of soil available for these studies, samples taken from C-34, C-35, and C-37 were homogeneously mixed and subsequently treated with two different final concentrations of TCE to assess the effect of TCE concentration on population counts and activities. TCE was added to soil in a glass beaker that was immediately covered and placed on ice to avoid TCE volatilization. This soil-TCE mixture was packed into columns using a wet packing method with intermittent vibration to exclude air bubbles. No pools of free DNAPL were obvious within the TCE-treated columns. The 1- and 5-week columns were treated with 40,000 mg TCE/kg soil (99.99%; Fisher Scientific, Pittsburgh, PA) to mimic the highest average concentrations observed at the source zone of the Sages site before ethanol flushing (Jawitz et al. 2000; Levine-Fricke-Recon, Inc. 1998). The 15-week columns were treated with 4,000 mg TCE/kg soil, which were concentrations reported at the Sages site by Sillan (1999).

After the columns were packed and TCE added, 10 pore volumes of groundwater taken from the Sages site were pumped at a flow rate of 0.2 mL/min using a Gilson peristaltic pump (Gilson, Inc., Middleton, WI). Two pore volumes of 70% ethanol were then pumped through the columns. After the ethanol flushing, 500 pore volumes of Sages site groundwater were pumped through the columns each week at a flow rate of 0.2 mL/min until each set of columns was sacrificed for genetic and activity analyses.

### Microbial DNA isolation.

Several methods to isolate DNA from the Sages site soil were attempted, as described in detail by Ramakrishnan (2002), including a commercial soil extraction kit marketed by Mo Bio Laboratories, Inc. (Solana Beach, CA) (used according to the manufacturer’s instructions) and modifications of previously reported methods (Berthelet et al. 1996; Cullen and Hirsch 1998; Duarte et al. 1998; Holben 1994; Miller et al. 1999; Ogram et al. 1987, 1997; Zhou et al. 1996).

DNA from enriched methanotrophic cultures was extracted using Mo Bio Laboratories DNA isolation kit. One and one-half milliliters of culture was centrifuged in Eppendorf tubes at 14,000 rpm for 2 min. Supernatant was discarded, and the pellet was added to the bead tube with buffer solution provided with the Mo Bio Laboratories kit. DNA from the cell pellet was isolated according to manufacturer’s instructions.

### Molecular analysis of soil and enriched culture DNA.

#### Polymerase chain reaction methods.

6S rRNA genes from bacterial and archaeal groups present in the soils were amplified by PCR, using specific primers. Universal bacterial primers used were 27F (AGAGTTTGATCCMTGGCTCAG) and 1492R (TACGGYTACCTTGTTACGAC TT) (Lane 1991). The reaction mixture contained 10 μL Hotstart mastermix (Qiagen, Valencia, CA), 1 μL (1 pmol) of each primer, and 8 μL soil DNA diluted at 1:10, 1:100, and 1:1,000. PCR was conducted using a PerkinElmer model 2400 DNA Thermal Cycler (PerkinElmer, Inc., Norwalk, CT) for 30 cycles with cycling parameters of 95°C for 15 min, followed by 94°C for 30 sec for denaturation of DNA, 58°C for 30 sec for annealing, and 72°C for 30 sec for DNA chain extension, followed by a 7-min chain extension step. Similarly, PCR was conducted with the universal archaeal primers 23F (TCYGGTTGATCCTGCC) (Burggraf et al. 1991) and 1492 R (Lane 1991) with cycling conditions similar to above. Reaction products were electrophoresed through a 0.7% agarose gel.

PCR of DNA isolated from methanotrophic enrichment cultures were performed with primers specific to 16S rRNA genes of type I and type II MTs. Primers used to amplify the 16S rRNA gene of type I MTs (labeled MT-I) were Meth T1dF 5′-CCTTCGGGMGCYGACGACT-3′ and Meth T1bR 5′-GATTCYMTGSATGT CAAGG-3′ (Wise et al. 1999). To amplify the 16S rRNA gene of type II MTs (labeled MT-II), universal bacterial primer 27 F (Lane 1991) and Meth T2R 5′-CATCTCTGRC SAYCATACCGG-3′ (Wise et al. 1999) were used. PCR primers used for sMMO were mmoX f882 (5′-GGCTCCAAGTTCAAG GTCGAGC-3′) and mmoX r1403 (5′ - T G G C A C T C G T A G C G C T C C G GCTCG-3′) (McDonald et al. 1995). Primers used for methanol dehydrogenase (MDH) were mxa f1003 (5′-GCGGCAC CAACTGGGGCTGGT-3′) and mxa r1561 (5′-GGGCAGCATGAAGGGCT CCC-3′) (McDonald and Murrell 1997). PCR was performed for 30 cycles in the PerkinElmer DNA model 2400 DNA Thermal Cycler previously described, with conditions of each reaction cycle held at 95°C for 15 min followed by 94°C for 30 sec (denaturation), 58°C for 30 sec (for type II primers), or 54°C for 30 sec (for type I primers) (annealing), and 72°C for 30 sec, with a final extension step at 72°C for 7 min (chain extension). Chromosomal DNA from *Methylosinus trichosporium* OB3b and *Methylomicrobium album* BG8 were used as positive controls for type II and type I PCR, respectively. PCR products were electrophoresed through a 0.7% agarose gel.

PCR analysis of sulfate reducers from soil enrichments was also performed. A 1.9-kb dissimilatory sulfite reductase (DSR) gene was amplified from cultures exhibiting sulfate-reducing activity using DSR1F (AC[C/G] CACTGGAACGACG) and DSR4R (GTG TACGACTTACCGCA) (Wagner et al. 1998) primers. PCR conditions were similar to those used for MTs mentioned previously, with the exception of an annealing temperature of 59°C for 30 sec and extension for 90 sec at 72°C.

#### Molecular cloning.

PCR products of approximately 1.5 kb were cloned using 5-min TA cloning kit (Invitrogen, San Diego, CA). The PCR product was ligated into the plasmid according to the manufacturer’s instructions. Two microliters of ligated plasmids were transformed to competent *Escherichia coli* cells (TOP10F′) provided with the cloning kit, followed by a heat shock at 42°C for 30 sec. *E. coli* cells were incubated at 37°C for 1 hr at 225 rpm with additional 250 μL SOC medium (2% tryptone, 0.5% yeast extract, 10 mM NaCl, 2.5 mM KCl, 10 mM MgCl_2_, 10 mM MgSO_4_, and 20 mM glucose). Transformed *E. coli* cells were plated onto Luria-Bertani (LB) plates with 50 μg/mL kanamycin and 40 μg/mL each X-gal (5-bromo-4-chloro-3-indolyl-β-d-galactoside or β-galactosidase) and IPTG (isopropylthio-β-d-galactoside) for screening of transformed cells. The plates were incubated overnight at 37°C. White colonies were randomly selected and inoculated into 5 mL LB-kanamycin broth (50 μg/mL) and incubated at 37°C overnight with shaking at 225 rpm.

#### Plasmid DNA isolation.

Twenty clones from most probable number clonings were randomly selected for screening. Plasmid DNA from cultures was isolated using a standard alkaline lysis procedure (Sambrook et al. 1989). *Eco*RI (Promega, Madison, WI) was used to digest plasmids to confirm whether the plasmids harbored PCR products. Isolated plasmids were digested overnight with 11 μL *Eco*RI, 1 μL 10× buffer, 6 μL deionized sterile water, and 2 μL plasmid DNA at 37°C. Digested products were electrophoresed through a 0.7% agarose gel. Inserts were digested with *Hha*I (Promega), electrophoresed as above, and grouped according to restriction fragment length polymorphism. Representatives of unique groups were selected for sequencing. The plasmid DNA was purified and inserts were sequenced by the Interdisciplinary Consortium for Biotechnology Research core sequencing facility at the University of Florida.

#### Acridine orange direct counting.

Soil samples (1 g wet weight) taken from various locations in the columns were preserved with 2.5% particle-free (0.2-μm pore-size filtered) glutaraldehyde. Samples were sonicated for 30 sec and kept on ice to avoid heating and damaging cells. The soil samples were then diluted 10-fold, and 100 μL of this suspension was poured into a 25-mm microfiltration system, equipped with a 0.2-μm polycarbonate filter (Isopore membrane filters; Millipore, Bedford, MA), and connected to a vacuum. To achieve random distribution of cells on the filter, the sample volume was increased to 2 mL with particle-free water (Turley 1993), and approximately 3 drops of acridine orange solution (1 mg/mL) were added to the sample. The filter unit was covered with aluminum foil to avoid photodegradation of acridine orange and was swirled for 3 min for random distribution and proper staining of cells. Samples were then filtered under a vacuum (Bio-Rad vacuum pump; Bio-Rad, Hercules, CA), with care taken not to allow drying of filter membranes. The damp filter membrane was placed on a clean glass slide with a fine smear of nonfluorescent immersion oil. A drop of immersion oil was placed on top of the filter membrane, and a cover slip was pressed firmly on the oil, with the oil forming a seal at the edge. Mounted slides were viewed under a 100× oil immersion objective of a Nikon Optiphot epifluorescent microscope (Nikon, Garden City, NY) fitted with filters for excitation of cells stained with acridine orange. Background counts were carried out with particle-free water, acridine orange, and glutaraldehyde solution and were subtracted from sample cell counts.

### Microbial activity measurements.

#### Methanogenic activity.

To compare the activity of methanogens in the columns, microcosms were constructed with 5 g nontreated starting soil and soil from the 1-, 5-, and 15-week column samples. Acetate (20 mM) or H_2_/CO_2_ (80:20%) (carbon/energy source) and sodium cysteine (pH 10.0) (reductant) were added to the soils in 60-mL vials that were incubated for 6 weeks at 28°C at 150 rpm.

Methane production was monitored by regular sampling and gas chromatography using a Hewlett-Packard model 5890 gas chromatograph (Hewlett-Packard, Denver, CO) equipped with a flame ionization detector and a 1/8 inch SS 45/60 Carboxen 1000 column. The temperatures of injector/detector and column were maintained at 110°C and 160°C, respectively. A standard gas (Scott Specialty Gases, Plumsteadville, PA) containing a mixture of 1% each of methane, carbon dioxide, carbon monoxide, oxygen, hydrogen, and the remainder nitrogen was used for standard curve calibration. Using gas-tight syringes (Hamilton, Reno, NV), 300 μL headspace gas was injected into the gas chromatograph.

#### Sulfate-reducing activity.

Sulfate-reducing microcosms were constructed using 5 g each of both Sages soil material (starting material) and column soil samples, using the same protocol described previously for enriching for these microorganisms. Vials were maintained at 28°C for 4 weeks. Dissolved sulfide concentrations were measured with a Shimadzu spectrophotometer (Shimadzu Biotech USA, Columbia, MD) at 480 nm, using the method described by Cord-Ruwisch (1985).

#### Methanotrophic activity.

Depletion of methane by 5 g of soil from the 1-, 5-, and 15-week columns was monitored in sealed microcosms constructed using the same protocol described previously for enriching for these microorganisms. Headspace sampling was performed regularly, followed by gas chromatographic analyses using a Hewlett-Packard model 5890 gas chromatograph equipped with a thermal conductivity detector, J&D Molesieve PLOT porous column (internal diameter, 20 m × 0.53 mm; Agilent/J&W Scientific, Palo Alto, CA), and a split/splitless injector. The temperatures of oven, injector, and detector were maintained at 25, 120, and 200°C, respectively. Head pressure was maintained at 5 psi. A certified grade 50/50 (methane/nitrogen) gas standard (Scott Specialty Gases, Inc.) was used for standard curve calibration. Initial rates of methane depletion were calculated using Excel 2000 software (Microsoft Corp., Redmond, WA) by determining the slopes of the resulting concentration-time plots using either linear or third-order polynomial fits, depending on the curvature of the methane depletion response. Standard errors of the initial slopes were determined using Simstat software (version 1.2.4e; Provalis Research, Ottawa, Ontario, Canada).

## Results and Discussion

### DNA isolation from Sages site soil samples.

As mentioned previously, various methods were employed to isolate amplifiable DNA from the Sages site soil samples to track changes in the microbial populations at the site with time. Regardless of the samples or methods used, however, extraction of microbial DNA from soil was problematic, as a deep brown substance co-purified with the DNA. Inability to isolate amplifiable DNA was attributed to co-purification of unknown PCR inhibitors with the DNA.

Amplifiable soil DNA was isolated only from samples MW-11 (taken immediately after flushing) and C-31 and C-35 (both taken 2 years after flushing). Results of PCR analysis showed the presence of bacterial and archaea genes in sample MW-11, only archaea genes in sample C-31, and only bacterial genes in sample C-33. No sample tested positive for the presence of the DSR gene or the type I or type II MT genes.

### Enrichments of sulfate-reducing and methanotrophic bacteria and screening for specific genes.

Given this difficulty in DNA isolation from the soil samples, enrichments of sulfate reducers and MTs were attempted from the Sages site soil as a means of verifying their presence and thus the potential for TCE transformation activity at the site. Positive results in these experiments would justify simulating ethanol flushing in the laboratory as an alternative means of tracking population changes in the soil over time.

Of the three Sages soil samples taken immediately after flushing, only cultures inoculated with MW-11 showed turbidity on repeated transfers and incubation at conditions conducive for methanotrophic growth, whereas five of the seven samples removed 2 years after flushing (C-31, C-33, C-35, C-36, and C-37) showed positive growth under these conditions. DNA isolated from each mixed methanotrophic–heterotrophic culture was subjected to PCR with primers specific to only type I and type II MTs (MT-I, MT-II), MDH and sMMO. Table 1 shows the results of these experiments, with presence or absence of the specific genes indicated by + and −, respectively. With the exception of the mixed culture derived from soil sample C-36, all other cultures showed the presence of not only universal bacterial genes but also genes specific for type II MTs, MDH, and sMMO. Interestingly, the location of soil sample C-36 is the closest to the ethanol-impacted area and would have naturally been exposed to the highest concentrations of ethanol and PCE.

A standard colorimetric naphthalene oxidation assay was performed to detect activity of sMMO in these methanotrophic–heterotrophic mixed cultures (Brusseau et al. 1990; Lindner et al. 2000). As shown in Table 1, the mixed cultures enriched from samples C-31, C-33, C-35, C-37, and MW-11 formed a purple color, whereas C-36 showed no color upon addition of tetrazotized *ortho*-dianisidine, known to complex with naphthol. These results are consistent with the results obtained from the PCR analysis confirming the presence of type II MT genes. Furthermore, the intensity of color formed was highest in C-37 when compared with the other samples.

The mixed cultures derived from soil samples were further screened by molecular cloning and 16S rRNA sequence analysis. The BLAST results of this analysis showing matches of the most similar GenBank sequences (GenBank 2002) are shown in Table 2. As shown, a type II MT sharing 98% 16S rRNA gene sequence similarity with *Methylocystis parvus* was identified in these mixed cultures.

Of the three soil samples taken immediately after flushing, only MW-11 yielded positive results for sulfate-reducing activities in the enrichments, as evidenced by a formation of a black precipitate of sulfide. Of those samples taken 2 years after flushing, only C-31 and C-37 yielded enrichment cultures of sulfate-reducing bacteria. As shown in Table 1, PCR analysis of these mixed cultures indicated the presence of DSR genes of all three of these samples. As observed in the methanotrophic enrichment experiments, the sample derived from C-36, closest to the ethanol-impacted area, yielded no indication of sulfate-reducing bacteria even 2 years after flushing.

### Column studies.

With the positive identification of methanotrophic and sulfate-reducing bacteria in some of the soil samples, column studies were performed with Sages site soil to simulate ethanol flushing and assess effects on microbial populations present over time. As described in “Materials and Methods,” columns were packed with soil derived from uncontaminated portions of the site, including sample C-37 that tested positively for both sulfate reducers and MTs.

#### Total microbial counts in soil columns.

**Figure 2:**
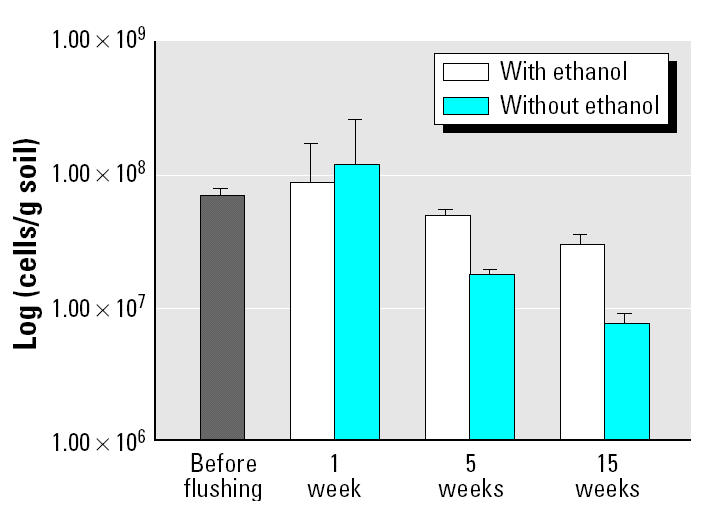
AODC of microbial cells in 1-, 5-, and 15-week column samples with and without ethanol flushing. “Before flushing” refers to Sages site soil from locations C-34, C-35, and C-37 used to pack the three columns. Error bars represent the 95% confidence interval of the average of seven samples.

Figure 2 shows that microbial counts in the soil column determined by acridine orange direct counting (AODC) methods did not significantly change 1 week after flushing with ethanol compared with counts in soils before flushing (8.63 × 10^7^ ± 2.22 × 10^7^ cells/g soil and 6.87 × 10^7^ ± 8.91 × 10^6^ cells/g soil, respectively). In addition, counts in columns with 40,000 mg TCE/kg soil only and no ethanol introduced (1.17 × 10^8^ ± 2.49 × 10^7^) were not significantly different from the ethanol-flushed column counts 1 week after flushing. However, soil removed from both flushed and nonflushed columns after 5 weeks possessed lower total counts than observed in the corresponding 1-week flushed columns. Surprisingly, these results imply that neither ethanol nor TCE immediately impacted the total microbial counts in the columns, but that these counts decreased in a 5-week period, with a greater decrease observed in the TCE-only columns. The 15-week columns pretreated with a significantly lower TCE concentration also showed higher total microbial counts in the flushed columns compared with the nonflushed columns but the lowest counts in comparison with the 1- and 5-week column cell numbers.

Although these results should be cautiously interpreted given the difficulties accompanying the AODC method (potential for human error, interference of humic acids, nonuniform distribution of microorganisms in the upflow columns), they do suggest that ethanol does not have the toxicity effects on the microorganisms as would be anticipated. In fact, higher counts observed in the flushed columns may indicate that it has a buffering effect on TCE toxicity to microorganisms.

### Activity measurements in column samples.

#### Methanogenic and sulfate-reducing activity.

In all microcosms incubated under methanogenic conditions, no methane formation was observed. On termination of the 5-week microcosms, a strong odor of hydrogen sulfide was noticed. As confirmation, dissolved sulfide was subsequently measured after 6 weeks in the microcosms established with soil from the 15-week columns. Despite no external sulfate added to these vials, hydrogen sulfide formation was observed, ranging from 6.75 ± 1.02 mM in the microcosms with soils treated with TCE and ethanol to 9.93 ± 2.36 mM with soils treated with ethanol only. The TCE-treated, nonflushed soils produced 8.25 ± 1.02 mM hydrogen sulfide, slightly lower than the ethanol-only treated columns. Continuous introduction of dissolved oxygen in the groundwater may have inhibited methanogenesis in the columns; however, channeling of the groundwater flow in the columns was observed, resulting in isolated regions that we suspected contained little or no oxygen. It was therefore anticipated that methanogenesis would have been detected in the activity assays.

Methanogenesis may also have been inhibited because of native sulfate concentrations (1.4–2.9 mg/L) that facilitated sulfate reduction as a primary process. Sulfate reducers compete with methanogens for carbon and energy sources, and because sulfate concentrations were relatively high in these soils, sulfate reduction was likely active in suppressing methanogenesis. Reduction in concentrations of PCE and accumulation of TCE and *cis*-dichloroethylene were observed at the Sages site after the ethanol treatment (Mravik et al. 2003; U.S. EPA 2000). This implies that reductive dehalogenation of PCE was taking place in the field. In fact, methane concentrations were reportedly increased at the Sages site in the flushing zone approximately 4 months after flushing and only after sulfate concentrations were depleted (Mravik et al. 2003). The microcosms using soils from the ethanol-only column produced 20–40% more sulfide compared with microcosms using soils from the columns treated with TCE only and with TCE and ethanol.

**Figure 3:**
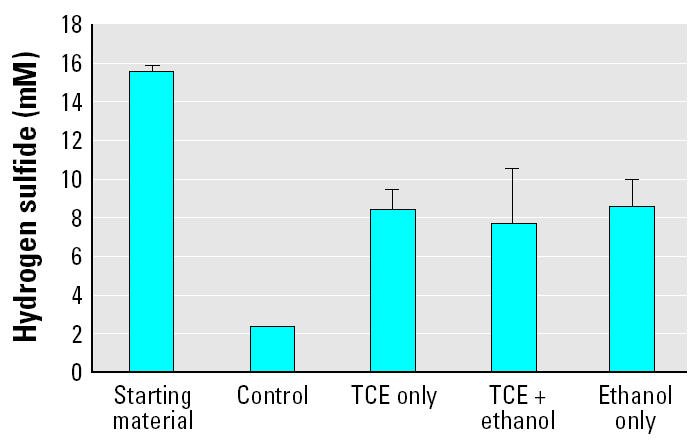
Hydrogen sulfide concentrations measured in sulfate-reducing microcosms using soils from the three 15-week columns tested in comparison with the starting material (not subjected to column treatments) and a control (no TCE or ethanol treatment). “TCE only” refers to microcosms with soils derived from columns pretreated with 4,000 mg TCE/kg soil with no ethanol flushing. “TCE + ethanol” refers to microcosms with soils taken from columns pre-treated with 4,000 mg TCE/kg soil and ethanol flushed. “Ethanol only” refers to microcosms with soils taken from columns with ethanol flushing and no TCE pretreatment. Error bars represent the 95% confidence interval of the average of a minimum of two measurements.

In the sulfide-reducing microcosms, where 20 mM sulfate was added to the microcosms, the starting material (with no treatment of TCE or ethanol) exhibited higher amounts of sulfide production (15.55 ± 0.35 mM) than the treated column samples after 15 weeks of groundwater flushing (Figure 3), implying an inhibitive effect of both ethanol and TCE. All microcosms with treated soils exhibited similar hydrogen sulfide production. The presence of residual ethanol after flushing did not affect sulfate-reducing activity (comparing all three 15-week column results). Ethanol may be used as an energy source by some sulfate reducers (Nagpal et al. 2000), but studies with postflushing samples from the Sages site have shown higher rates of PCE dechlorination with whey as electron donor rather than with ethanol (Helton 2000).

#### Methanotrophic activity.

Microcosms were established with soils from the 1- and 5-week columns and incubated under a 20% initial headspace concentration of methane to assess methane depletion activities over time. An initial lag period of 1–2 days was observed in all 1- and 5-week microcosms. Table 3 shows the resulting initial rates of methane depletion and percentage of total methane removed during the 11-day testing period, after which no noticeable change in methane concentrations was observed. Rates of methane depletion were significantly higher (2.17% headspace methane removed per day) with the soils in the 5-week columns treated with TCE only compared with the 1-week column soils (0.83% headspace methane removed/day). The 5-week soils dosed with only TCE also removed a greater total percentage of methane during the experiments than the 1-week soils (45 and 17%, respectively). Little difference was observed in the methane depletion activities in the microcosms constructed with soils removed from the 1- and 5-week TCE- and ethanol-treated columns, as shown in Table 3. However, in comparison with the TCE-only soils, both “TCE + ethanol” soils (1-week and 5-week) showed much higher initial methane depletion rates. These soils treated with TCE and ethanol in the 1-week columns displayed a higher percentage removal of methane (37%) compared with the corresponding soils treated with TCE only (17%). The total methane removed by the 5-week soils treated with TCE with or without ethanol was not significantly different from the total amount of methane removed by the 1-week soils, however.

The higher methane depletion activities observed in soils 1 and 5 weeks after ethanol flushing compared with non–ethanol-treated soils indicate that ethanol has a mitigating effect on TCE toxicity to methanotrophic bacteria. The toxicity of products of TCE metabolism by MTs, including TCE epoxide, has been previously reported (Alvarez-Cohen and McCarty 1991; Oldenhuis et al. 1991; Oldenhuis and Janssen 1993), and the fact that no methanotrophic enrichments were successful at the sampling location closest to the ethanol flushing may be attributed to the higher concentrations of PCE and TCE present and not of ethanol.

The 15-week column soils showed little difference in methane depletion rates and total methane removed regardless of the treatment subjected to the soils, and no lag period was observed in any of the microcosms. In each of these microcosms, 84–88% of methane was removed, which was significantly higher than observed in the 1- and 5-week microcosms. Although it is tempting to compare these values to those of the 1- and 5-week column soils, caution should be taken in doing so because of the large difference in initial TCE dose used (40,000 mg TCE/kg soil in the 1- and 5-week columns; 4,000 mg TCE/kg soil in the 15-week columns). What can be concluded from these results, however, is that even 15 weeks after ethanol flushing, methanotrophic activity potential is present in the soils, as well as the potential for removal of residual TCE, given appropriate conditions at a treatment site.

## Conclusions

Because the DNA isolation was problematic, it was not possible to test samples directly at the site as originally planned. However, the study was successful in enriching for sulfate-reducing bacteria and type II methanotrophic bacteria. Additionally, the column studies showed that no methanogenesis occurred, possibly because of the predominance of the sulfate-reducing activity, in agreement with observations taken during the pilot-scale flushing event.

The goal of this work was to determine if the introduction of the ethanol during flushing impacted the activities (and indirectly, their ability to transform residual contaminant) of the microorganisms present in the Sages site soil. Total counts of bacteria decreased in all flushed and nonflushed samples with time; however, flushed samples contained higher total counts of bacteria compared with those in nonflushed samples. Sulfide formation was observed not only in sulfate-reducing microcosms with soils from the 15-week laboratory columns and initially dosed with 4,000 mg TCE/kg soil, but also in methanogenic microcosms in as little as 1 week. Methanotrophic activity potentials increased from 1 to 5 weeks, and little difference in methane depletion was observed with the 15-week soils regardless of treatment. However, higher rates of methane depletion were observed in those microcosms with the 1- and 5-week column soils subjected to ethanol flushing. These results indicate that ethanol flushing did not have as severe an impact on the populations as was initially anticipated and did not impair the activities of the sulfate-reducing and methanotrophic microorganisms over time. Furthermore, increased activity observed in the presence of ethanol indicates the mitigating effects of ethanol to TCE toxicity.

## Figures and Tables

**Table 1:** PCR analysis and sMMO assay results of mixed methanotrophic–heterotrophic enrichments.

Sample	Bacteria	Archaea	DSR	MT-I	MT-II	MDH	sMMO	sMMO assay
MW-11	+	−	+	−	+	+	+	+
C-31A	+	−	+	−	+	+	+	+
C-33A	+	−	−	−	+	+	+	+
C-35A	+	−	−	−	+	+	+	+
C-36A	−	−	−	−	−	−	−	−
C-37A	+	−	+	−	+	+	+	+

Presence or absence of genes or positive or negative assay results denoted by + or −, respectively.

**Table 2:** BLAST results of 16S rDNA sequence analysis mixed methanotrophic cultures.

Most similar GenBank sequence*^a^*	Identity (%)	GenBank accession number*^a^*
*M. parvus*	98	AF150805
*Acinetobacter calcoaceticus*	99	AF159045
*Acaligenes* sp.	98	AF150805
*Hypomicrobium facilis*	99	Y14311

a From GenBank (2002).

**Table 3:** Initial methane depletion rates and total percentage of methane depleted observed in the methanotrophic microcosms using 1-, 5-, and 15-week soil samples.

	TCE alone			TCE + ethanol			
Activity measure	1 week	5 weeks	15 weeks	1 week	5 weeks	15 weeks	Ethanol alone 15 weeks
Methane depletion rate (% CH_4_/day)	0.83 (0.19)	2.17 (0.40)	4.03 (2.61)	14.61 (4.27)	14.14 (9.68)	4.37 (0.56)	5.09 (0.33)
Percentage of total methane removed	17	45	84	37	43	87	88

Numbers in parentheses denote the standard error on the slopes of the lines used to calculate the initial methane depletion rates.
